# Altered resting-state neural activity and changes following a craving behavioral intervention for Internet gaming disorder

**DOI:** 10.1038/srep28109

**Published:** 2016-07-06

**Authors:** Jin-Tao Zhang, Yuan-Wei Yao, Marc N. Potenza, Cui-Cui Xia, Jing Lan, Lu Liu, Ling-Jiao Wang, Ben Liu, Shan-Shan Ma, Xiao-Yi Fang

**Affiliations:** 1 State Key Laboratory of Cognitive Neuroscience and Learning and IDG/McGovern Institute for Brain Research, Beijing Normal University, Beijing 100875, China; 2 Center for Collaboration and Innovation in Brain and Learning Sciences, Beijing Normal University, Beijing 100875, China; 3 Departments of Psychiatry and Neuroscience, Child Study Center, and CASAColumbia, Yale University School of Medicine, New Haven, CT 06519, USA; 4 Connecticut Mental Health Center, New Haven, CT 06519, USA; 5 Students Counseling Center, Beijing Normal University, Beijing 100875, China; 6 Institute of Developmental Psychology, Beijing Normal University, Beijing 100875, China

## Abstract

Internet gaming disorder (IGD) has become a serious mental health issue worldwide. Evaluating the benefits of interventions for IGD is of great significance. Thirty-six young adults with IGD and 19 healthy comparison (HC) subjects were recruited and underwent resting-state fMRI scanning. Twenty IGD subjects participated in a group craving behavioral intervention (CBI) and were scanned before and after the intervention. The remaining 16 IGD subjects did not receive an intervention. The results showed that IGD subjects showed decreased amplitude of low fluctuation in the orbital frontal cortex and posterior cingulate cortex, and exhibited increased resting-state functional connectivity between the posterior cingulate cortex and dorsolateral prefrontal cortex, compared with HC subjects. Compared with IGD subjects who did not receive the intervention, those receiving CBI demonstrated significantly reduced resting-state functional connectivity between the: (1) orbital frontal cortex with hippocampus/parahippocampal gyrus; and, (2) posterior cingulate cortex with supplementary motor area, precentral gyrus, and postcentral gyrus. These findings suggest that IGD is associated with abnormal resting-state neural activity in reward-related, default mode and executive control networks. Thus, the CBI may exert effects by reducing interactions between regions within a reward-related network, and across the default mode and executive control networks.

Internet gaming disorder (IGD), which was included in section 3 of the Diagnostic and Statistical Manual of Mental disorder, 5^th^ Edition as a topic deserving more research^1^, has become a serious mental health issue worldwide^2^. Since IGD may jeopardize individuals’ health, academic performance, and interpersonal relationships, evaluating interventions for IGD, as well as their mechanisms of action, is of great significance^3^.

As existing data suggest similarities in brain structure and function between IGD and substance and gambling addictions^4^,^5^. IGD is regarded as a behavioral addiction^2^,^6^. Investigating neurobiological factors related to IGD would not only advance our understanding of its underlying mechanism, but also provide information to develop more effective interventions^7^,^8^. However, the effects of interventions on neural activity in IGD remain largely unknown.

Resting-state fMRI has emerged as a powerful tool to investigate intrinsic brain activity that is not confounded by task performance^9^ and has been used to investigate various psychiatric disorders, including IGD^10^,^11^,^12^. Differences in resting-state functional connectivity (rsFC) have been linked to IGD including brain regions within or between the default mode network (DMN)^13^,^14^, executive control network (ECN)^11^,^12^, and reward network^11^,^15^,^16^,^17^. In addition to these rsFC findings, studies measuring other indices such as amplitude of low fluctuation (ALFF)^18^ have indicated that IGD is associated with altered resting-state neural activity in regions involved in reward processing and attention, including the orbitofrontal cortex (OFC) and precuneus^10^.

Compared with traditional self-report indices, resting-state fMRI may offer a more objective indicator^19^. Although no study has directly evaluated how interventions for IGD may alter resting-state neural activity, several studies have explored this issue in substance addictions. Specifically, Konova *et al*.^19^ found that a single dose of oral methyphenidate “normalized” altered functional connectivity within reward network in patients with cocaine addiction. Sutherland *et al*.^20^ found that varenicline and nicotine could decrease rsFC of the insula with amygdala and components of the DMN in abstinent smokers, and Schmaal *et al*.^21^ found that modafinil decreased the interactions between the ECN and DMN in alcohol-dependent patients. Another study showed that brief meditation training not only reduced tobacco use and self-reported craving, but also increased ALFF in the anterior cingulate and prefrontal cortex, regions involved in self-control^22^. Therefore, meditation training may improve self-control through increasing frontal cortical regional spontaneous activity or/and connectivity to suppress craving, ultimately reducing addictive behaviors^22^.

As mentioned above, the rsFC and ALFF are two widely used measures in resting-state fMRI studies. ALFF focuses on the regional intensity of spontaneous fluctuations^18^, whereas rsFC reflects interactions between brain regions^9^. Since these two approaches reflect different profiles of intrinsic brain activity and largely complement each other^23^, a study that combines ALFF and rsFC would further elucidate neural differences at rest and, in conjunction with therapeutic intervention data, and provide insight into neural mechanisms underlying treatments for IGD.

The present study aimed to combine ALFF and rsFC approaches to identify abnormal intrinsic neural activity in IGD, and how a craving behavioral intervention (CBI), an intervention for addictive disorders^7^,^24^, may operate at a neural level. Although no existing study has directly examined neural mechanisms of interventions for IGD, considering the similarities between IGD and other addictive disorders^2^,^5^,^6^, we hypothesized that, compared with healthy comparison (HC) subjects, individuals with IGD would demonstrate altered resting-state activity in regions involved in reward processing, and regions responsible for attention and cognitive control within the DMN and ECN. We also hypothesized that abnormal regional resting-state activity and interactions between these brain areas would be altered in IGD subjects receiving CBI^19^,^21^,^22^,^24^.

## Materials and Methods

### Participants

This study was part of a larger study of developing and evaluating an effective psychobehavioral intervention for IGD, in which 63 IGD and 22 HC subjects were included, and 44 IGD and 22 HC subjects participated in resting-state fMRI scanning based on their willingness and suitability for fMRI. In the present study, we focused only subjects who participated in resting-state fMRI scanning.

Participants were recruited by means of online advertisements and word of mouth and were selected through an online questionnaire and telephone screening. Only right-handed male participants were included because of the higher prevalence of IGD in men versus women^25^. Eight IGD and 3 HC subjects were excluded due to excessive head motion during scanning. Thus, data from 36 IGD subjects and 19 HC subjects were included for final analyses in the present study.

Participants were recruited according to their weekly Internet gaming time and scores on the Chen Internet Addiction Scale^26^, which consists of 26 items on a 4-point Likert scale. Inclusion criteria for IGD subjects were: 1) a score of 67 or higher on the CIAS^25^; 2) engagement in Internet gaming for over 20 hours per week for a minimum of one year; and 3) reporting of Internet gaming as their primary online activity^17^,^27^. Inclusion criteria for HC subjects were: 1) a score of 60 or lower on the CIAS; and 2) less than 2 hours per week spent on Internet games. Moreover, all participants were assessed by a semi-structured personal interview, and anyone who met any of the following criteria was excluded: 1) current or history of use of illegal substances and gambling; 2) current or prior psychiatric or neurological illness; and 3) current use of psychotropic medications.

Among 36 IGD subjects, 20 (CBI + group) were willing to participate in group CBI once a week, lasting for six weeks, and were scanned before and after the intervention. The remaining 16 IGD subjects (CBI– group) did not receive any intervention, but were similarly scanned at the same time points.

This study complied with the Declaration of Helsinki and was approved by the Institutional Review Board of the State Key Laboratory of Cognitive Neuroscience and Learning, Beijing Normal University. All participants provided written informed consent and were financially compensated for their time.

### Assessment

Internet gaming characteristics were assessed by the CIAS^26^, weekly gaming time, and the brief questionnaire of Internet (gaming) craving, an 8-item 7-point Likert scale adapted from the Questionnaire of Smoking Urges^28^.

Cigarettes and alcohol use were recorded, and the Fagerstrom Test for Nicotine Dependence^29^ and the alcohol consumption questions from the Alcohol Use Disorders Identification Test^30^ were used to assess tobacco and alcohol use disorders, respectively. Additionally, current status of depression and anxiety were assessed using the Beck Depression Inventory^31^ and the Beck Anxiety Inventory^32^.

### Craving Behavioral Intervention (CBI)

The CBI was developed on the basis of behavioral intervention^7^,^24^, the craving framework of boundary conditions^33^, and the fulfillment of psychological needs for Internet use^34^, Since craving may play a critical role in the development and maintenance of IGD, interventions that help subjects to cope with and reduce craving may promote positive therapeutic outcomes and prevent relapse^7^,^35^. The group intervention was conducted once a week, including six 2.5-hour sessions, with 8–9 IGD subjects in each group. The topic for each session was: 1) perceiving subjective craving and mindfulness training for gaming-cue-induced craving and tension; 2) recognizing and testing irrational beliefs regarding craving; 3) detecting craving and training in mindfulness to relieve craving-related negative emotions; 4) training on coping with cravings and altering participants’ fulfillment of psychological needs; 5) learning time management and skills training for coping with craving; 6) reviewing, practicing, and implementing skills. The details of the CBI are included in the supplementary materials.

### MRI Data Acquisition and Preprocessing

Resting-state fMRI data were obtained on a 3.0 T Siemens Trio scanner at Brain Imaging Center, Beijing Normal University. Participants were instructed to keep their head still and eyes open during scanning. Earplugs and ahead coil with foam pads were used to minimize machine noise and head motion. Scanning parameters were: repetition time = 2000 ms, echo time = 30 ms, flip angle = 90°, field of view = 200 × 200 mm^2^, acquisition matrix = 64 × 64, voxel size = 3.1 × 3.1 × 3.5 mm^3^, slice = 33, time point = 200. A T1-wighted scan was acquired with following parameters: repetition time = 2530 ms, echo time = 3.39 ms, flip angle = 7°, field of view = 256 × 256 mm^2^, voxel size = 1 × 1 × 1.33 mm^3^, slice = 144.

Data were preprocessed and analyzed using DPABI version 1.2 (http://rfmri.org/dpabi)and SPM8 software package (http://www.fil.ion.ucl.ac.uk/spm). The first 10 volumes were discarded to allow the magnetization to approach a dynamic equilibrium and to allow participants to get used to the scanning noise. Individual EPI data were slice-time corrected. Participants whose head motion exceeding 3.0 mm in translation or 3° in rotation (8 IGD subjects and 3 HC subjects) were excluded. We further reduced potential confounds of head motion with Friston-24 correction at the individual level and mean framewise displacement (FD)^36^ regression at the group level^37^,^38^. Since head motion may impact rsFC results even after these regressions, we confirmed our rsFC analyses using FD scrubbing correction, in which time points with head motion exceeding 0.5 mm were excluded from the analyses^36^,^38^. To reduce possible effects of physiological artifacts, we covaried signals from cerebrospinal fluid and white matter^39^. EPI data were then normalized to the Montreal Neurological Institute (MNI) space. A spatial filter of 4 mm full width at half maximum Gaussian kernel was used. Subsequently, a band pass temporal filter (0.01–0.10 Hz) was applied to reduce the low-frequency drifts and high-frequency noise^20^.

### ALFF Calculation

ALFF calculation was performed similarly as in previous studies^10^,^18^, using REST version 1.8^40^. The filtered time series was transformed to the frequency domain through fast Fourier transform. Then the square root of the power spectrum was obtained for each frequency and the averaged square root was obtained across 0.01–0.10 Hz at each voxel, and the averaged square root was taken as the ALFF measurement. The ALFF of each voxel was divided by the global mean of ALFF for standardization. A two-sample *t*-test was conducted to compare whole-brain ALFF between IGD and HC subjects.

### rsFC Calculations

Two seeds, the right OFC (x = 27, y = 48, z = 3), and posterior cingulate cortex (PCC; x = 6, y = −30, z = 33), were selected on the basis of ALFF comparison between IGD and HC subjects. Seeds were 6 mm in radius. The average time-series within each seed were regressed against whole-brain voxels to generate cross correlation maps. Correlation coefficient images were Fisher Z-transformed.

### Statistical Analysis

For behavioral data, firstly, independent-sample t-tests for continuous variables or chi-square tests for categorical variables were used for between-group comparisons of the demographic and Internet gaming characteristics (i.e., CIAS scores, weekly gaming time, craving). Secondly, analyses of variance (ANOVAs) with repeated measures were used to examine the effects of CBI on Internet gaming characteristics, with group (CBI + and CBI−) as a between-subject factor, and session (baseline and second test) as a within-subject factor.

For fMRI data, we first compared baseline ALFF and rsFC between IGD subjects and HC subjects to explore altered intrinsic neural activity in IGD subjects. A group-difference map was corrected by means of Gaussian random field theory (voxel-wise: minimum Z score >2.3; cluster significance: *P* < 0.05), resulting in a family-wise error (FWE) rate < 5%.

To examine the effects of CBI on IGD subjects, we compared ALFF and rsFC changes ([ALFF or rsFC at the second scanning]–[ALFF or rsFC at baseline]) between CBI + and CBI− groups (FWE-corrected).

Finally, Pearson correlation analyses were conducted to examine the associations between the Internet gaming characteristics and resting-state neural activity identified by the two-sample *t*-tests in the IGD group. Additionally, the associations between changes of Internet-gaming characteristics and changes of resting-state neural activity were examined in the CBI + group using Pearson correlation analyses.

## Results

### Demographics and Internet Gaming Characteristics of IGD and HC subjects

IGD subjects and HC subjects did not differ in age, education, or alcohol use and cigarette smoking measures. As expected, IGD subjects reported higher CIAS and craving scores, and showed higher anxiety and depression symptoms, in comparison to HC subjects. Since head motion may influence on rsFC results, we also compared the mean FD between IGD and HC subjects and found no group differences in this measure of head motion (Table S1).

### Baseline ALFF and rsFC Differences between IGD and HC subjects

**Figure 1:**
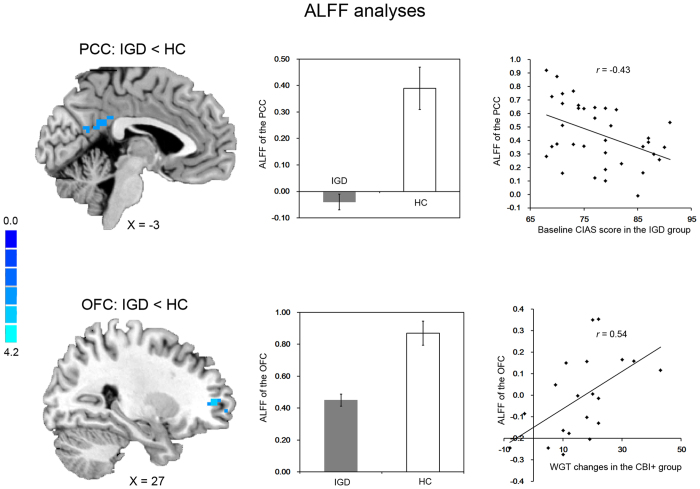
ALFF of the OFC and PCC in IGD and HC subjects and associations with behavioral measures. ALFF, amplitude of low fluctuation; OFC, orbital frontal cortex; PCC, posterior cingulate cortex; IGD, Internet gaming disorder; HC, healthy comparison.

IGD subjects showed significantly lower baseline ALFF in the right OFC and PCC compared with HC subjects (Fig. 1 and Table 1). We further compared rsFC of these two regions between IGD and HC groups. IGD subjects showed significantly higher rsFC between the PCC and right dorsolateral prefrontal cortex (DLPFC). However, no between-group differences on OFC-centered rsFC were observed.

### Demographics and Internet Gaming Characteristics of CBI + and CBI– groups

As shown in Table 2, the CBI + and CBI– groups did not differed significantly on demographics. With regard to Internet gaming characteristics, similar results were found in ANOVAs with repeated measures on CIAS (main effect of session: *F*_(1,34)_ = 63.05, *P* < 0.001; main effect of group: *F*_(1,34)_ = 2.67, *P* = 0.11; interaction effect: *F*_(1,34)_ = 26.60, *P* < 0.001), weekly gaming time (main effect of session: *F*_(1,34)_ = 9.45, *P* = 0.004; main effect of group: *F*_(1,34)_ = 2.75, *P* = 0.11; interaction effect: *F*_(1,34)_ = 7.07, *P* = 0.012), and craving (main effect of session: *F*_(1,34)_ = 98.146, *P* < 0.001; main effect of group: *F*_(1,34)_ = 2.23, *P* = 0.15; interaction effect: *F*_(1,34)_ = 3.94, *P* = 0.055). Simple-effect analyses for group indicated that CBI + and CBI– groups did not differed significantly on baseline CIAS (*F*_(1,34)_ = 3.70, *P* = 0.06), and weekly gaming time (*F*_(1,34)_ = 1.05, *P* = 0.31), but the CBI + group scored significantly lower than did the CBI– group on the second-test measures (*F*_(1,34)_ = 14.40, *P* = 0.001 for CIAS, *F*_(1,34)_ = 7.18, *P* = 0.011 for weekly gaming time).

### Changes in resting-state neural activity in the CBI + and CBI– groups

**Figure 2:**
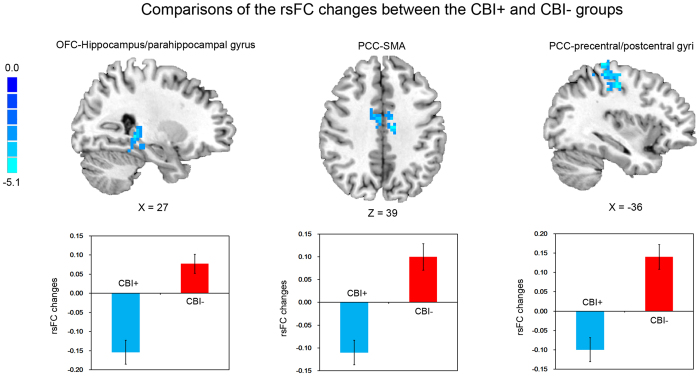
Comparisons of the rsFC changes ([rsFC at the second scanning]–[rsFC at baseline]) between the CBI + and CBI– groups. rsFC, resting-state functional connectivity; CBI + , participants who received craving behavioral intervention; CBI–, participants who did not receive craving behavioral intervention; OFC, orbital frontal cortex; PCC, posterior cingulate cortex; SMA, supplementary motor area.

We compared the ALFF and rsFC of the right OFC and PCC between the CBI + and CBI– groups and no between-group differences in the ALFF changes were observed. However, compared with the CBI– group and after receiving CBI, the CBI + group showed significantly reduced rsFC of the: (1) OFC with a cluster containing the hippocampus and parahippocampal gyrus, and (2) PCC with the supplementary motor area, precentral gyrus, and postcentral gyrus (Fig. 2 and Table 1).

### Brain-behavior Relationships

ALFF of the PCC was negatively associated with CIAS scores across all IGD subjects (*r* = −0.43, *P* = 0.008; Fig. 1). In addition, although no significant associations between changes of the resting-state neural activity and Internet-gaming characteristics were observed in the CBI + group, the ALFF in the OFC at baseline was positively associated with changes in weekly gaming time (*r* = 0.54, *P* = 0.015) in the CBI + group.

## Discussion

The present study combined ALFF and rsFC measures to investigate differences between IGD and HC subjects and how CBI may operate to alter ALFF and rsFC. IGD subjects demonstrated lower ALFF in the OFC and PCC, and higher interactions between the PCC and DLPFC, relative to HC subjects. Moreover, compared with the CBI–group, the CBI + group showed reduced rsFC of the OFC with the hippocampus and parahippocampal gyrus, and reduced rsFC of the PCC with the supplementary motor area, precentral gyrus, and postcentral gyrus. These results suggest that resting-state neural activity in reward-related network, DMN and ECN are dysfunctional in IGD. Moreover, CBI appears to alter the functional organization and communication of these networks in IGD.

We observed reduced ALFF in the right OFC in IGD subjects, compared with HC subjects. The OFC contributes to the integrating and evaluating of rewards^8^,^41^ and is important for not only craving^42^,^43^, but also decision-making^44^,^45^. The current finding is consistent with previous observations that the ALFF of the OFC was decreased in chronic heroin users^46^, and suggests the OFC may be a potential therapeutic target across addictive disorders. Consistent with this viewpoint, we found that CBI reduced the interactions between the OFC and a cluster containing the hippocampus and parahippocampal gyrus in the CBI + group. The hippocampus and parahippocampal gyrus also belong to a reward-related network^41^, with the hippocampus involved in the processing of contextual reward-related information, and the parahippocampal gyrus involved in cue-induced craving in IGD^4^. Taken together, these findings suggest the altered intrinsic activity in the OFC may be a hallmark in IGD, and CBI may exert effects by reducing the functional connectivity of the OFC with other regions responsible for reward processing, such as the hippocampus and parahippocampal gyrus.

We also observed reduced ALFF in the PCC in IGD subjects compared with HC subjects. In addition, IGD subjects demonstrated significantly higher rsFC of the PCC with the right DLPFC, relative to HC subjects. These findings are consistent with a body of research indicating that spontaneous activity and interactions with other brain regions in the PCC are dysfunctional in individuals with IGD^10^,^13^,^14^. The PCC is a key component in the DMN, contributing importantly to self-monitoring and attention^9^,^47^, whereas the DLPFC is involved in the ECN^48^. The DMN and ECN are two competitive brain networks, with a generally negative coupling between these two networks^49^,^50^. However, heightened communications between components of the DMN and ECN, which are typically regarded as maladaptive, have been previously reported in addictive disorders^9^,^21^. The present study, together with previous findings mentioned above, suggests that abnormal intrinsic activity within the DMN, and heightened interactions between the DMN and ECN, may serve as candidate neural markers of IGD.

Although pathological ALFF in the PCC or rsFC between the PCC and DLPFC was not directly normalized in the CBI + group after receiving intervention, the interactions between the PCC and supplementary motor area, precentral and postcentral gyri was significantly decreased in CBI + group. These regions, especially the supplementary motor area and precentral gyrus, largely belong to the ECN^48^. These findings suggest that CBI may operate through reducing the interactions between components of the DMN and ECN, paralleling modafinil-induced effects on intrinsic neural activity in alcohol dependent patients^21^. The competitive relationship between networks involved in externally and internally oriented cognition was reorganized in CBI + group, and such changes in the intrinsic organization of the brain may improve cognitive control, which has been described as being critical for reducing craving and addictive behaviors^22^.

We further examined relationships between Internet gaming characteristics and the abnormal intrinsic activity of the PCC and OFC within the IGD group and found that IGD subjects with higher IGD severity demonstrated lower ALFF in the PCC. This finding further suggests dysfunction of the PCC in IGD. In addition, the ALFF in the OFC at baseline was positively associated with reduction in weekly gaming time in CBI + group, indicating that IGD subjects with higher ALFF in the OFC before intervention achieved better intervention effects. Future interventions for IGD combining CBI and other approaches, such as transcranial magnetic stimulation (TMS)^51^,^52^ or real-time fMRI neurofeedback^53^, to increase the spontaneous activity within the OFC may significantly improve intervention outcomes; however, this speculation warrants direct investigation.

The present study also provides empirical support to theoretical models of IGD. For example, Brand *et al*. proposed that IGD is characterized by enhanced craving for gaming and related cues which is related to function in the reward-related regions such as the OFC and weakened executive control which is related to function in the executive control network such as the DLPFC^35^. In addition, a cognitive-behavioral model also hypothesizes that motivational drives related to reward-seeking and cognitive control related to executive inhibition are two critical domains that are impaired in IGD and may serve as promising targets for interventions^7^. Our findings are largely consistent with these two models of IGD. Furthermore, previous theories mainly focused in dysfunctions on reward or executive system. The present study indicates that maladaptive interactions between networks may be as important as alterations in specific brain regions. Future theoretical models of IGD should consider this condition from a network-based prospective.

The findings of the present study should be interpreted in the light of its limitations. First, although IGD is most prevalent in young adult males^25^,^54^, our recruitment of only this population limits the generalizability of our findings. Second, we failed to identify any significant associations between changes in Internet gaming characteristics and intrinsic neural activity. Future studies should collect more IGD-related variables, such as cognitive factors underlying IGD, to further explore potential mechanisms of CBI. Third, IGD subjects were not randomly assigned to CBI + or CBI– group; rather assignment was based on the willingness of subjects. Moreover, the CBI + and CBI– groups were not matched, with the CBI + group scoring marginally higher on the baseline CIAS and marginally lower on the BAI. After adding these scores as covariates in the ANOVAs with repeated measures, the interaction effect on CIAS remained and the interaction effects on weekly gaming time and Internet gaming craving failed to reach statistical significance (see Table S2 for details). Therefore, the current findings should be regarded as preliminary and should be confirmed in randomized controlled trials. Finally, the CBI– group did not receive any specific intervention. For this reason, potential confounds related to possible expectations regarding behavioral and neural changes cannot be excluded. Thus, our findings should be further verified by studies with an active control group (e.g., relaxation training).

In summary, the present study indicates that IGD is associated with differences in regional resting-state activity or maladaptive rsFC in the reward-related, default mode and executive control networks. Furthermore, interactions between regions within reward-related network, and interaction between the DMN and ECN were decreased in CBI + group after receiving intervention. Collectively, this study provides new insights in possible neurobiological mechanisms of CBI on intrinsic neural activity in IGD subjects and has significant implications for the development of more effective interventions.

## Additional Information

**How to cite this article**: Zhang, J.-T. *et al*. Altered resting-state neural activity and changes following a craving behavioral intervention for Internet gaming disorder. *Sci. Rep.* **6**, 28109; doi: 10.1038/srep28109 (2016).

## Supplementary Material

Supplementary Information

## Figures and Tables

**Table 1:** ALFF and rsFC analyses results.

	Brain region	Side	BA	Voxels	T	x	y	z
Baseline ALFF: HC>IGD	OFC	R	10	57	3.96	27	48	3
	PCC	R/L	23/31	51	3.62	6	−30	33
Baseline rsFC: IGD>HC	PCC-DLPFC	R	46	120	4.43	42	45	27
rsFC (second scanning – baseline): CBI–>CBI+	OFC-Hippocampus/parahippocampal gyrus	R		85	4.86	27	−36	−3
	PCC-SMA/ACC	L	6/24	268	5.07	−12	−15	39
	PCC-precentral/postcentral gyri	L	3/4	232	4.41	−36	−33	69

IGD = Internet gaming disorder; HC = healthy comparison; CBI: craving behavioral intervention; ALFF: amplitude of low fluctuation; rsFC: resting-state functional connectivity; OFC: orbital frontal cortex; PCC: posterior cingulate cortex; DLPFC: dorsolateral prefrontal cortex; SMA: supplementary motor area; ACC: anterior cingulate cortex; R: right; L: left; BA: Brodmann area.

**Table 2:** Demographics and Internet-gaming Characteristics of the CBI + and CBI– groups.

	CBI + (*n* = 20)	CBI– (*n* = 16)	*t* value	*P*
	mean ± S.D.	mean ± S.D.		
Age	21.80 ± 1.70	22.38 ± 1.71	−1.01	0.32
Years of education	15.90 ± 1.41	15.44 ± 1.82	0.86	0.40
BAI	3.65 ± 3.58	7.88 ± 7.27	−2.05	0.053
BDI	9.15 ± 6.05	10.44 ± 4.26	−0.75	0.46
Baseline CIAS	79.70 ± 6.55	75.38 ± 6.90	1.92	0.06
CIAS at the second test	59.70 ± 9.22	71.13 ± 8.66	−3.79	0.001
Baseline weekly gaming time	28.58 ± 10.62	25.31 ± 7.87	1.02	0.31
Weekly gaming time at the second test	12.20 ± 8.07	24.13 ± 17.79	−2.68	0.01
Baseline craving for gaming	38.80 ± 6.93	38.00 ± 7.27	0.34	0.74
Craving for gaming at the second test	17.65 ± 5.81	23.88 ± 9.95	−2.22	0.04
Baseline FD	0.15 ± 0.08	0.13 ± 0.05	0.92	0.37
FD at the second test	0.12 ± 0.06	0.11 ± 0.04	0.91	0.37

S.D. = standard deviation; CBI: craving behavioral intervention; CIAS = Chen Internet addition scale; BAI = Beck Anxiety Inventory; BDI = Beck Depression Inventory; FD = framewise displacement.
