# Supplementary material for: Altered resting-state neural activity and changes following a craving behavioral intervention for Internet gaming disorder
Source: Sci Rep. 2016 Jul 6;6:28109. doi: 10.1038/srep28109 (PMC4933876; doi:10.1038/srep28109)
Supplement: Supplementary Information [file srep28109-s1.doc]

**Altered resting-state neural activity and changes following a craving behavioral intervention for Internet gaming disorder**

Jin-Tao Zhang^1,2^, Yuan-Wei Yao^1^, Marc N. Potenza^3,4^, Cui-Cui Xia^5^, Jing Lan^6^, Lu Liu^6^, Ling-Jiao Wang^1^, Ben Liu^1^, Shan-Shan Ma^1^, Xiao-Yi Fang^6*^

^1^ State Key Laboratory of Cognitive Neuroscience and Learning and IDG/McGovern Institute for Brain Research, Beijing Normal University, Beijing 100875, China

^2^ Center for Collaboration and Innovation in Brain and Learning Sciences, Beijing Normal University, Beijing 100875, China

^3^ Departments of Psychiatry and Neuroscience, Child Study Center, and CASAColumbia, Yale University School of Medicine, New Haven, CT 06519, USA

^4^ Connecticut Mental Health Center, New Haven, CT 06519, USA

^5^ Students Counseling Center, Beijing Normal University, Beijing 100875, China

^6^ Institute of Developmental Psychology, Beijing Normal University, Beijing 100875, China

*Corresponding author: Xiao-Yi Fang, Tel: 8610-5880-8232, Fax: 8610-5880-8232, E-mail address: fangxy@bnu.edu.cn

**Methods**

*Craving behavioral intervention (CBI)*

The CBI for Internet gaming disorder (IGD) was developed on the basis of behavioral intervention (Dong and Potenza, 2014), the craving framework of boundary conditions (McCarthy et al., 2010), and the fulfillment of psychological needs for Internet use (Suler, 1999). The CBI was developed to help individuals with IGD to: 1) recognize subjective craving and its relationship with Internet gaming behaviors; 2) reduce craving through ameliorating the salience of gaming-cues and craving-related irrational beliefs, withdrawal symptoms and other negative affects; 3) enhance self-monitoring and control for craving through time management training; and, 4) relieve fulﬁllment of psychological needs through Internet use and attenuate the relationship between craving and gaming behaviors through coping skill training.

The CBI was given once a week for 6 weeks, conducted by four therapists with similar clinical background with respect to training in behavioral therapy and group therapy. A pair of therapists was randomly assigned to a CBI+ group. Each session included 5 parts in 2.5-3 hours: a warming-up exercise, a discussion about the homework from the last session (except the ﬁrst session), a main structured activity, a brief summary, and the homework assignment.

The topics for each session focused on: 1) understanding and perceiving subjective craving for Internet gaming, listing gaming-related scenes that might trigger craving, and mindfulness training for gaming-cue-induced craving and tension; 2) recognizing and testing irrational beliefs regarding craving and exploring other possible inferences; 3) detecting emotions following craving and searching effective experience of craving regulation; mindfulness training to experience and relieve craving-related emotions; 4) shifting participants' fulﬁllment of psychological needs from the Internet to reality and building adaptive relationships with peers; 5) learning time management and skills training for coping with craving; 6) maintaining the effectiveness of the intervention through reviewing and practicing, and setting up adaptive and positive plans for daily life in the future. In addition, mindfulness training was self-administered every time they experienced craving outside the intervention hours as an assignment.

**Additional results**

Table S1

Demographics and Internet Gaming Characteristics of IGD and HC subjects.

|  | IGD subjects  (*n* = 36) | HC subjects  (*n* = 19) | *t*/χ^2^ value | *P* |
| --- | --- | --- | --- | --- |
|  | mean ±S.D. | mean ±S.D. |  |  |
| Age (in years) | 22.06 ± 1.71 | 22.89 ± 2.23 | -1.56 | 0.13 |
| Years of education | 15.69 ± 1.60 | 16.57 ± 1.98 | -1.79 | 0.08 |
| CIAS | 77.78 ± 6.96 | 42.11 ± 8.27 | 16.93 | < 0.001 |
| Weekly gaming time (in hours) | 27.13 ± 9.51 | 1.67 ± 0.58 (n=3) | 15.72 | < 0.001 |
| Craving for Internet (gaming) | 38.44 ± 6.99 | 13.00 ± 4.18 | 16.87 | < 0.001 |
| Alcohol use  (at least once per month) | 26/36 | 13/19 | 0.09 | 0.77 |
| AUDIT-C | 3.12 ± 1.80 (n=26) | 2.23 ± 1.17 (n=13) | 1.61 | 0.12 |
| Cigarette use  (at least once per month) | 2/36 | 0/19 | - | - |
| FTND | 2.29 ± 1.50 | - | - | - |
| BAI | 5.53 ± 6.01 | 2.00 ± 3.27 | 2.79 | 0.008 |
| BDI | 9.72 ± 5.29 | 2.78 ± 4.33 | 4.81 | < 0.001 |
| Baseline FD | 0.14 ± 0.07 | 0.13 ± 0.05 | 0.55 | 0.59 |

IGD = Internet gaming disorder; HC = healthy control; S.D. = standard deviation; CIAS = Chen Internet addition scale; AUDIT-C = alcohol consumption questions from the Alcohol Use Disorders Identification Test; FTND = Fagerstrom test for nicotine dependence; BAI = Beck Anxiety Inventory; BDI = Beck Depression Inventory; FD = framewise displacement.

Table S2

Results of ANOVAs with repeated measures on CIAS, weekly gaming time, and craving after controlling for baseline CIAS and BAI.

| Dependent variable | Main effect of group | Main effect of session | Interaction effect |
| --- | --- | --- | --- |
| CIAS | *F*_(1,32)_=3.67, *P*=0.06 | *F*_(1,32)_=12.31, *P*=0.001 | *F*_(1,32)_=12.31, *P*=0.001 |
| Weekly gaming time | *F*_(1,32)_=3.00, *P*=0.09 | *F*_(1,32)_=2.26, *P*=0.14 | *F*_(1,32)_=2.42, *P*=0.13 |
| Internet gaming craving | *F*_(1,32)_=0.91, *P*=0.35 | *F*_(1,32)_=0.74, *P*=0.40 | *F*_(1,32)_=1.08, *P*=0.31 |

Table S3

rsFC analyses results after scrubbing correction

|  | Brain region | Side | BA | Voxels | T | x | y | z |
| --- | --- | --- | --- | --- | --- | --- | --- | --- |
| Baseline rsFC: IGD > HC | PCC-DLPFC | R | 10/46 | 189 | 4.47 | 48 | 48 | 9 |
|  | PCC-DLPFC | L | 9/46 | 163 | 4.49 | -39 | 36 | 36 |
| rsFC (second scanning – baseline):  CBI– > CBI+ | OFC-Hippocampus/parahippocampal gyrus | R |  | 145 | 5.85 | 27 | -36 | -3 |
|  | PCC-SMA/precentral/postcentral gyrus | L | 3/4/6 | 469 | 4.29 | -36 | -33 | 69 |
|  | PCC-cerebellum posterior lobe | R |  | 115 | 3.95 | 12 | -60 | -51 |

IGD = Internet gaming disorder; HC = healthy control; CBI: craving behavioral intervention; ALFF: amplitude of low fluctuation; rsFC: resting-state functional connectivity; OFC: orbital frontal cortex; PCC: posterior cingulate cortex; DLPFC: dorsolateral prefrontal cortex; SMA: supplementary motor area; R: right; L: left; BA: Brodmann area.

**Reference**

Dong G, Lin X, Potenza MN (2015b) Decreased functional connectivity in an executive control network is related to impaired executive function in Internet gaming disorder. Prog Neuro-Psychopharmacol Biol Psychiatry 57:76-85.

McCarthy DE, Curtin JJ, Piper ME, Baker TB (2010) Negative reinforcement: Possible clinical implications of an integrative model. In: Kassel JD, editor. Substance abuse and emotion. Washington, DC: American Psychological Association. p 15-42.

Suler JR (1999) To get what you need: healthy and pathological Internet use. CyberPsychol Behav 2:385-393.
